# Accuracy of an HRP-2/panLDH rapid diagnostic test to detect peripheral and placental *Plasmodium falciparum* infection in Papua New Guinean women with anaemia or suspected malaria

**DOI:** 10.1186/s12936-015-0927-5

**Published:** 2015-10-19

**Authors:** Alexandra J. Umbers, Holger W. Unger, Anna Rosanas-Urgell, Regina A. Wangnapi, Johanna H. Kattenberg, Shadrach Jally, Selina Silim, Elvin Lufele, Stephan Karl, Maria Ome-Kaius, Leanne J. Robinson, Stephen J. Rogerson, Ivo Mueller

**Affiliations:** Department of Medicine at the Doherty Institute, The University of Melbourne, Melbourne, Australia; Papua New Guinea Institute of Medical Research (PNG IMR), Goroka, Papua New Guinea; Walter and Eliza Hall Institute of Medical Research (WEHI), Melbourne, Australia; Barcelona Institute for Global Health (ISGLOBAL), Barcelona, Spain; Institute of Tropical Medicine, Antwerp, Belgium

**Keywords:** Sensitivity, Specificity, *Plasmodium*, Pregnancy

## Abstract

**Background:**

The diagnosis of malaria during pregnancy is complicated by placental sequestration, asymptomatic infection, and low-density peripheral parasitaemia. Where intermittent preventive treatment (IPT) with sulfadoxine-pyrimethamine is threatened by drug resistance, or is inappropriate due to low transmission, intermittent screening and treatment (ISTp) with rapid diagnostic tests for malaria (RDT) could be a valuable alternative. Therefore, the accuracy of RDTs to detect peripheral and placental infection was assessed in a declining transmission setting in Papua New Guinea (PNG).

**Methods:**

The performance of a combination RDT detecting histidine-rich protein-2 (HRP-2) and *Plasmodium* lactate dehydrogenase (pLDH), and light microscopy (LM), to diagnose peripheral *Plasmodium falciparum* and *Plasmodium vivax* infections during pregnancy, were assessed using quantitative real-time PCR (qPCR) as the reference standard. Participants in a malaria prevention trial in PNG with a haemoglobin ≤90 g/L, or symptoms suggestive of malaria, were tested. Ability of RDT and LM to detect active placental infection on histology was evaluated in some participants.

**Results:**

Among 876 women, 1162 RDTs were undertaken (anaemia: 854 [73.5 %], suspected malaria: 308 [26.5 %]). qPCR detected peripheral infection during 190 RDT episodes (165 *P. falciparum*, 19 *P. viva*x, 6 mixed infections). Overall, RDT detected peripheral *P. falciparum* infection with 45.6 % sensitivity (95 % CI 38.0–53.4), a specificity of 96.4 % (95.0–97.4), a positive predictive value of 68.4 % (59.1–76.8), and a negative predictive value of 91.1 % (89.2–92.8). RDT performance to detect *P. falciparum* was inferior to LM, more so amongst anaemic women (18.6 vs 45.3 % sensitivity, Liddell’s exact test, *P* < 0.001) compared to symptomatic women (72.9 vs 82.4 % sensitivity, *P* = 0.077). RDT and LM missed 88.0 % (22/25) and 76.0 % (19/25) of *P. vivax* infections, respectively. In a subset of women tested at delivery and who had placental histology (n = 158) active placental infection was present in 19.6 %: all three peripheral blood infection detection methods (RDT, LM, qPCR) missed >50 % of these infections.

**Conclusions:**

In PNG, HRP-2/pLDH RDTs may be useful to diagnose peripheral *P. falciparum* infections in symptomatic pregnant women. However, they are not sufficiently sensitive for use in intermittent screening amongst asymptomatic (anaemic) women. These findings have implications for the management of malaria in pregnancy. The adverse impact of infections undetected by RDT or LM on pregnancy outcomes needs further evaluation.

**Electronic supplementary material:**

The online version of this article (doi:10.1186/s12936-015-0927-5) contains supplementary material, which is available to authorized users.

## Background

Malaria during pregnancy is a preventable and treatable disease, which remains responsible for an enormous health, social and economic burden for communities in the developing world. Malaria infection is a major threat to maternal and neonatal survival, resulting in up to 200,000 infant deaths and 10,000 maternal deaths each year [1]. Globally 125 million pregnancies remain at risk of malaria exposure [2], three quarters of which occur in the Asia–Pacific region.

Diagnosing malarial infection in pregnancy is challenging. In areas of high endemicity the majority of infected women are asymptomatic [3]. Diagnosis is further complicated by low peripheral blood parasite densities and sequestration of *Plasmodium falciparum*-infected erythrocytes in the placental intervillous space, termed placental malaria (PM) [4]. Antenatal detection of PM in particular is difficult [5], yet it is PM that is principally associated with a number of severe adverse pregnancy outcomes, including low birthweight (LBW) [4]. Intermittent preventive treatment in pregnancy with sulfadoxine-pyrimethamine (SP-IPTp) provides regular parasite clearance and partial chemoprophylaxis in the absence of knowledge of infection status [6], overcoming some of the aforementioned diagnostic challenges. However, this intervention is threatened by drug resistance [7, 8], and may be inappropriate in low or unstable transmission settings [9], and as such, is currently not endorsed by the World Health Organization (WHO) for use outside of sub-Saharan Africa [6].

Recently, there has been considerable interest in intermittent screening and treatment in pregnancy (ISTp) as an alternative to SP-IPTp, in particular in areas where high-level drug resistance is a concern [10]. This approach consists of regular antenatal screening events using rapid diagnostic tests (RDTs): they are accessible, affordable and require little operational expertise. Results of the first reported trial of ISTp, which was conducted in an area of moderately high malaria transmission in Ghana, suggest that ISTp using an lactate dehydrogenase-based RDT prevents LBW and severe anaemia to a similar extent to SP-IPTp [10], however the impact on PM was not assessed. Currently available RDTs are known to miss a substantial number of peripheral and placental infections [11]: as such, the findings of the Ghana trial indicate that infections undetectable by RDT may not be an important cause of maternal anaemia and LBW [10]. This contrasts findings of other research suggesting a role of ‘sub-RDT’ and ‘submicroscopic’ *P. falciparum* infections in causing adverse pregnancy outcomes, in particular anaemia, in some [5, 12–14], but not all [15, 16], studies evaluating these infections.

RDTs perform well outside of pregnancy and the WHO recommends their use for the diagnosis of malaria in this context [17]. Less is known about their accuracy in pregnancy, in particular when polymerase chain reaction (PCR) and placental histology are used as reference (‘gold standard’) [11]. Moreover, there is a paucity of information regarding the utility of RDTs for the management and prevention of malaria in pregnancy in the Asia–Pacific region, where both *P. falciparum* and *P. vivax* frequently co-exist [18–20] and cause poor pregnancy outcomes [21]. In Papua New Guinea (PNG), gestational malarial infection is common and frequently associated with adverse pregnancy outcomes [22, 23]. PNG national guidelines recommend RDTs and light microscopy (LM) to diagnose infection in symptomatic patients, including pregnant women [24]. Given all human malaria species, bar *Plasmodium knowlesi,* are sympatric in PNG, combination RDTs detecting both *P. falciparum*-histidine-rich-protein-2 (HRP-2) and the genus-specific malaria antigen lactate dehydrogenase (pLDH) are most appropriate, and were shown to be appropriate for malaria treatment amongst febrile children in PNG [25]. To date, the performance characteristics of these RDTs in pregnant women in PNG remain unknown.

This study evaluated the accuracy of an HRP2/pLDH combination RDT and LM to diagnose peripheral *P. falciparum* and *P. vivax* infections as detected by qPCR in the context of a large clinical trial of malaria prevention in pregnancy in PNG. Secondary objectives included an assessment of RDT performance characteristics relative to qPCR amongst both women with asymptomatic anaemia vs those with suspected malaria, and an evaluation of the performance of peripheral blood RDT, LM and qPCR to detect placental infection as observed on histological examination.

## Methods

### Study setting and design

This study was conducted between July 2010 and November 2013 in a prospective cohort of pregnant women enrolled in a clinical trial evaluating three doses of IPTp with SP plus azithromycin vs a single dose SP plus chloroquine and two placebo doses from second trimester in PNG (NCT01136850) [26]. The trial was conducted at nine health centres in Madang Province on the North Coast of PNG. The study area was previously considered hyperendemic for *P. falciparum* and *P. vivax* [27]. Prevalence of malaria in pregnancy fell considerably over the 5 years prior to, and spanning, the study period [28].

During the original trial passive case detection forms (morbidity episodes) were completed for women who reported new or recent illness or where found to be anaemic. The trial was designed such that an RDT was performed during a morbidity episode when malaria was suspected clinically or when women were found to be moderately or severely anaemic (“anaemia”, defined here as a haemoglobin measurement (Hb) <90 g/L for moderate, and <60 g/L for severe (HemoCue Ltd, Angelholm, Sweden, accuracy of 1 g/L) and/or presence of pallor. Because women living in lowland PNG were highly likely to be at least mildly anaemic by WHO standards [29], a more conservative Hb cut-off for ‘anaemia’ was used to refine eligibility. Malaria was suspected if women had one or more of the following: fever (≥37.5 °C), history of fever (within previous 48 h), headache, chills and rigors, and joint or muscle pain. The choice of testing criteria for suspected malaria was based on previous research on symptoms associated with malaria in pregnancy in areas considered hyperendemic for *P. falciparum* [30, 31].

Accuracy of the RDT, and LM, to detect peripheral *P. falciparum* and *P. vivax* parasitaemia was assessed using qPCR as the Ref. [32]. A subset of trial participants had both an RDT and LM performed within 12 h of birth and placental histology assessed: this permitted an evaluation of the performance of peripheral blood RDT, LM and qPCR to detect active placental malaria.

### Study participants

Pregnant women presenting for their first antenatal visit were screened according to inclusion criteria of the original trial [26]. In brief, women were required to be <26 gestational weeks by abdominal palpation, ≥16 years of age, without severe symptomatic anaemia (Hb <60 g/L), and without permanent disability or chronic medical conditions. HIV status of participants was not determined as antenatal HIV prevalence at the provincial hospital (Modilon General Hospital) was 1.1 % (2009–2012, unpublished audit data). Trial participants who had one or more RDT done for aforementioned test criteria, and who had complete data for LM and qPCR for at least one RDT screening episode, were eligible for inclusion in the present study.

### Sampling and laboratory procedures

Malaria was diagnosed using the commercially available three-band CareStart™ P.f/Pan combo RDT (Access Bio, USA, Lot MR1J3, Exp Feb 2014), which is pre-coated with two monoclonal antibodies, one detecting *P. falciparum*-specific malaria antigen HRP-2, the other genus-specific LDH. Using LM as reference, the manufacturer reported sensitivities of 98 and 96 % to detect peripheral *P. falciparum* and *P. vivax* infections, respectively in non-pregnant individuals [33].

Testing was undertaken by trained clinical research staff, and test kits were stored and performed according to the manufacturer’s instructions [33]. There were no invalid test results. Women with positive RDTs were treated for malaria according to national guidelines (quinine in first trimester, artemether-lumefantrine thereafter) [24].

Peripheral blood smears were prepared, and venous blood samples taken, each time an RDT was performed. Labeled blood smears were air-dried and stained with 4 % Giemsa for 30 min. Thick smears were used to count the number of asexual parasites per 200 leukocytes (or per 500 if <10 parasites/200 leukocytes), assuming 8000 leukocytes/µL of blood; slides were declared negative if no parasite was seen in 200 oil-immersion fields. Two microscopists read each slide, and third reads were performed to resolve discrepant results [34]. If species discrepancies were not resolved by this third read (5/1162 RDT screening episodes), qPCR results were used and considered definite [32]. The microscopy laboratory at the PNG Institute of Medical Research undergoes regular and external quality control.

qPCR was performed to detect *P. falciparum* and *P. vivax* infections in peripheral and placental blood samples using an established methodology with a sensitivity of 1 parasite per microliter [35]. *Plasmodium ovale* and *Plasmodium malariae* infections in pregnant PNG women are rare [36], and budgetary constraints precluded their evaluation.

A subset of women had placental tissue collected for histological processing and analysis as described previously [37]. Placenta malaria was diagnosed and staged according to three histological features: presence of infected erythrocytes, malaria pigment in monocytes/macrophages, and malaria pigment in fibrin deposits [38, 39]. Active placental malaria was diagnosed upon detection of parasites.

Readers of all aforementioned the index tests (LM and RDT) and respective reference standards (qPCR, or placental histology) were blinded to the results of the other tests.

### Definitions

qPCR was used as the reference method for the detection of peripheral parasitaemia, and placental histology to identify women with active placental infection. All active placental infections were assumed to be due to *P. falciparum*: *P. vivax* has been associated with placental changes but these differ markedly from *P. falciparum* and are rare [40].

An RDT was classified positive for *P. falciparum* if showing (a) a positive reaction at the HRP-2 band, or (b) positive reactions at both the HRP-2 band and pLDH band. The RDT was classified positive for *P. vivax* if showing a positive reaction at the pLDH band only, or at both the HRP-2 band and pLDH band (possible mixed infection).

### Statistical analysis

Data were double entered into a study databases (FoxPro 9.0 or Excel, Microsoft, USA) and analysed using Stata 12.0 (StataCorp, USA). Univariate comparisons of variables were undertaken using the Chi-squared and Fisher’s exact test for binary data, as appropriate, and the Student’s *t* test and the Mann–Whitney-*U* test for parametric data and nonparametric data, respectively.

Sensitivity, specificity, positive predictive value (PPV) and negative predictive value (NPV) with 95 % confidence intervals were calculated for the comparisons of RDT against qPCR, and LM against qPCR, for the detection of peripheral *P. falciparum* parasitaemia, and RDT, LM and qPCR of peripheral blood for the detection of active placental infection. Analysis of diagnostic performance of RDT and LM to detect *P. vivax* infection was restricted to sensitivities, as qPCR did not include *P. ovale* and *P. malariae.* In addition, performance of RDT to detect malarial infection (any species) observed on LM (the current reference standard in PNG) was assessed. Sensitivities and specificities were compared using Liddell’s exact test for pairwise comparisons of proportions [41, 42]. Parasite densities were presented as geometric means. All analyses used α < 0.05 as the cut-off for statistical significance. A sample size calculation was performed for the original trial but not for the purpose of the present sub-analysis [26]. The study was reported according to STARD guidelines (Standards for the reporting of diagnostic accuracy studies).

### Ethical considerations

Written informed consent was obtained from all participating women. Study procedures were in accordance with good clinical and laboratory practice. Ethical approval for study protocol was obtained from the PNG Institute of Medical Research Institutional Review Board (0815), the PNG Medical Research Advisory Council (08.01), and the Melbourne Health Human Research Ethics Committee (2008.162). The trial was registered with the United States National Institutes of Health Clinical Trials Registry (NCT01136850) and has been reported according to STARD guidelines.

## Results

**Fig. 1:**
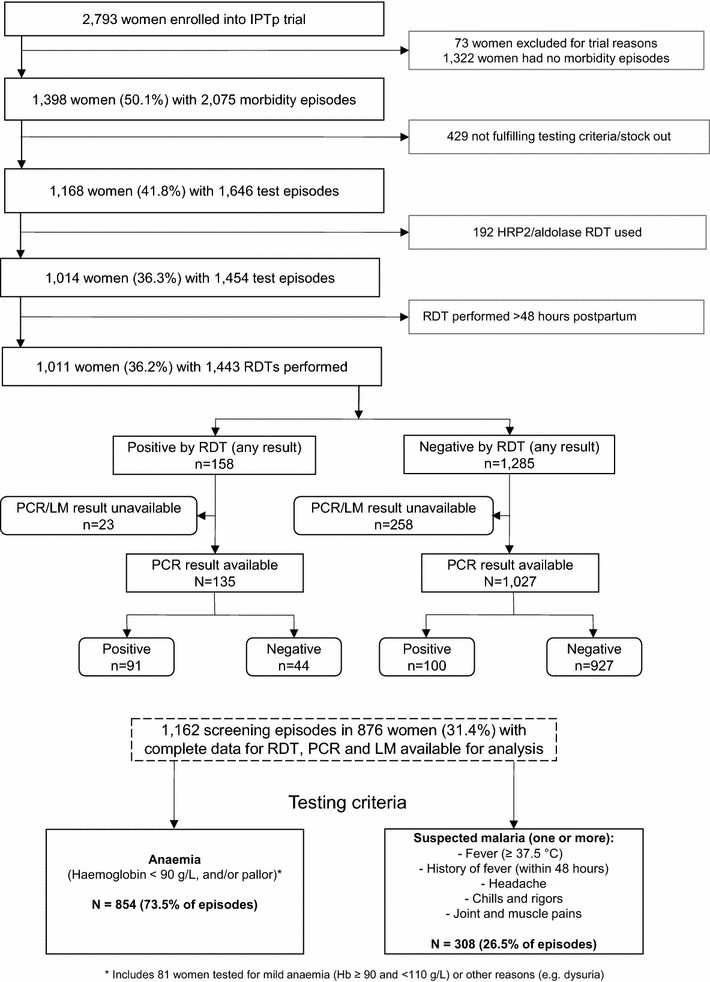
Study flow diagram illustrating RDT test exclusion criteria, tests performed with matched PCR and LM availability, by testing criteria for anemia or suspected malaria

Of 2793 women enrolled in the original trial, 876 (31.4 %) women met the criteria for RDT testing at least once during pregnancy, had complete data for peripheral blood RDT, LM and qPCR, and were thus eligible for inclusion in the present analysis. The remaining 1917 were either withdrawn from the original study (n = 73, 2.6 %), did not meet testing criteria (n = 1322, 47.3 %), or had incomplete test results (n = 522, 18.7 %; Fig. 1). Background characteristics of women who were tested solely for anaemia and those who had symptoms were similar (Table 1).

**Table 1:** Participant (n = 876) and episode (n = 1162) characteristics, by testing criteria

Characteristic	All (n = 876)	Anaemia only* (n = 639)	Symptomatic (n = 237)	*P*
Participant				
Age	24.6 ± 5.4	24.5 ± 5.4	24.8 ± 5.5	0.446
Primigravida	48.3 (423)	47.3 (302)	51.1 (121)	0.318
Fundal height at enrolment	22 [6–27]	22 [6–27]	21 [6–27]	0.002
Rural dweller	70.6 (618)	70.9 (453)	69.6 (165)	0.714
SPAZ-IPTp	49.9 (437)	51.6 (330)	45.2 (107)	0.088

	All (n = 1162)	Anaemia only* (n = 854)	Symptomatic (n = 308)	
Episode				
RDT				
HRP2 +	5.3 (62)	2.1 (18)	14.3 (44)	<0.001
HRP2 + pLDH +	4.5 (52)	1.2 (10)	13.6 (42)	<0.001
pLDH +	1.8 (21)	0.8 (7)	4.5 (14)	<0.001
Light microscopy				
*P. falciparum*	11.5 (133)	5.6 (48)	27.6 (85)	<0.001
Asexual parasite density	5141 (1437; 16–123,484)	2237 (441; 39–19,372)	6731 (2418; 16–123,484)	0.001
<200 parasites/µL	21.1 (28/133)	29.2 (14/48)	16.5 (14/85)	0.085
*P. vivax*	0.9 (10)	0.9 (8)	0.7 (2)	0.480
Asexual parasite density	434 (96; 16–3341)	470 (60; 16–3341)	288 (288; 143–432)	<0.001
<200 parasites/µL	80.0 (8/10)	87.5 (7/8)	50.0 (1/2)	0.378
qPCR positive				
P. falciparum**	14.7 (171)	10.1 (86)	27.6 (85)	<0.001
P. vivax**	2.2 (25)	2.5 (21)	1.3 (4)	0.358

Descriptive data on participant pregnancy characteristics of all women tested and by those tested specifically for anaemia or symptomatic malaria criteria. Descriptive data of rapid diagnostic test results, light microscopy and qPCR by all women tested, and and by those tested specifically for anaemia or symptomatic malaria criteria. Numbers are mean ± standard deviation, median [range], geometric mean (median; range) or  % (n). SPAZ-IPTp, intermittent preventive treatment with sulfadoxine-pyrimethamine plus azithromycin; HRP2, histidine-rich protein 2; pLDH, genus-specific lactate dehydrogenase

* Includes 81 women that were tested for mild anaemia (Hb ≥90 and <110 g/L) or for other reasons

** 6 women had mixed *P. falciparum/P. vivax* infections

Amongst participating women (n = 876) a total of 1162 tests were performed. One quarter of women (25.7 %, 225) had two or more RDT screening episodes: the mean (median, range) number of days between testing episodes was 57 (42, 11–206) days. A quarter of tests were performed because women presented with symptoms suggestive of malaria (26.5 % of episodes, n = 308) (Table 1, Fig. 1). Four women had two positive RDT results (over 60 days apart), despite receiving malaria treatment between positive tests. These women either had a negative RDT episode in between positive RDT episodes, or had infections caused by two different species (according to qPCR), making antigen persistence an unlikely reason for the positive second test.

qPCR detected peripheral infection during 190 (16.4 %) RDT screening episodes (165 *P. falciparum*, 19 *P. viva*x, 6 *P. falciparum*/*P. vivax* mixed infections) (Table 1). 11.6 % (n = 135) RDT screening episodes were positive by RDT, including 114 for HRP-2. A total of 143 episodes (12.3 %) were positive by LM (133 *P. falciparum*, 10 *P. vivax*). Compared with women tested due to anaemia, women who presented with symptoms of suspected malaria were more likely to have a positive RDT and to have *P. falciparum* infection diagnosed by LM or qPCR, and had higher parasite densities when they were infected (Table 1).

Overall, RDT missed 54.4 % of peripheral *P. falciparum* detected by qPCR (93/171), and a higher proportion (81.4 %, 70/86) of infections were missed in women who were asymptomatic (Table 2). LM failed to detect a third of *P. falciparum* infections observed by qPCR, yet had higher sensitivity than RDT, particularly amongst asymptomatic anaemic women (45.3 % [38.0, 53.4] *vs* 18.6 % [11.0-28.4], Liddell’s exact test, *P* < 0.001) (Table 2). Both RDT and LM were highly specific, although LM had slightly better specificity for *P. falciparum* amongst symptomatic women compared to RDT (93.3 *vs* 89.2 %, Liddell’s exact test, *P* = 0.035) (Table 2). There were 22 HRP-2 positive RDT screening episodes that were negative for *P. falciparum* by both LM and qPCR: qPCR threshold cycle and parasitaemia characteristics of discrepant cases are presented in Additional file 1: Table S1. Diagnostic performance indicators of RDT and LM for the detection of *P. falciparum* by gravidity and trimester are presented in Additional file 2: Table S2.

**Table 2:** Comparison of HRP2/pLDH RDT (and light microscopy) against qPCR (reference) for detection of *P. falciparum* and *P. vivax* infection in peripheral blood (n = 1162)

Category/test	Prevalence by qPCR (95 % CI)	Sensitivity (95 % CI)	Specificity (95 % CI)	PPV (95 % CI)	NPV (95 % CI)	Infections missed (per 1000 women)
P. falciparum						
Overall						
RDT	14.7 (12.7, 16.9)	45.6 (38.0, 53.4)**	96.4 (95.0, 97.4)	68.4 (59.1, 76.8)	91.1 (89.2, 92.8)	93 of 171 (54.4 %)
LM		63.7 (56.1, 70.9)**	97.6 (96.4, 98.4)	82.0 (74.4, 88.1)	94.0 (92.3, 95.4)	62 of 171 (36.3 %)
Anaemia only (n = 854)						
RDT	10.1 (8.1, 12.3)	18.6 (11.0, 28.4)**	98.4 (97.3, 99.2)	57.1 (37.2, 75.5)	91.5 (89.4, 93.3)	70 of 86 (81.4 %)
LM		45.3 (34.6, 56.5)**	98.8 (97.8, 99.5)	81.3 (67.4, 91.1)	94.2 (92.3, 95.7)	47 of 86 (54.7 %)
Symptomatic (n = 308)						
RDT	27.6 (22.6, 32.6)	72.9 (62.2, 82.0)	89.2 (84.4, 93.0)*	72.1 (61.4, 81.2)	89.6 (84.9, 93.3)	23 of 85 (27.1 %)
LM		82.4 (72.6, 89.8)	93.3 (89.1, 96.2)*	82.4 (72.6, 89.8)	93.3 (89.1, 96.2)	15 of 85 (17.6 %)
P. vivax						
RDT	2.2 (1.3, 3.2)	12.0 (2.6, 31.5)	–	–	–	22 of 25 (88.0 %)
LM		24.0 (9.4, 45.1)	–	–	–	19 of 25 (76.0 %)

Performance characteristics of RDT against qPCR for detection of infection by species in peripheral blood samples. *RDT* rapid diagnostic test, *LM* light microscopy, *qPCR* real-time polymerase chain reaction, *PPV* positive predictive value, *NPV* negative predictive value. Sensitivity was defined as the proportion of true positives (RDT or LM positive) from all matched positive qPCR samples while specificity was defined as the proportion of true negatives (RDT or LM negative results) of all matched qPCR negative samples. Positive predictive value was defined as how frequently the RDT or LM tested positive in matched qPCR positive samples, while negative predictive value was defined as the frequency of RDT or LM testing negative in matched qPCR negative samples

* Liddell’s exact test P < 0.05

** Liddell’s exact test P < 0.001

Prevalence of peripheral *P. vivax* infection by qPCR was low (n = 25); although LM missed more than three quarter of infections it detected twice as many compared to RDT (Table 2). Of 22 *P. vivax* infections missed by RDT nine were detected by LM (four of them were classified as *P. falciparum* by LM). The geometric mean density of these infections was 157 parasites/μL (median 125, range 16–3341), with seven out of nine infections exhibiting a parasite density <200 parasites/μL. Specificities were not assessed as the RDT measured pan-specific LDH and qPCR for *P. ovale* and *P. malariae* were not performed.

A total of 49 of 143 (34.3 %) infections of any species detected by LM remained undetected by the HRP-2/pLDH RDT. Geometric mean density of these infections was 278 parasites/μL (median 220, range 16–14,717; one infection gametocytes seen only). Specifically, RDT had a sensitivity of 66.0 % (95 % CI 57.6–73.7), specificity of 96.1 % (94.7–97.2), PPV of 70.4 % (61.9, 77.9) and NPV of 95.2 % (93.7–96.4) to detect microscopic peripheral parasitaemia.

A quarter of women (221/876) had an RDT performed at delivery. Of these 71.5 % (n = 158) had a placental biopsy taken for histological evaluation of placental malaria, and 31 (19.6 %) had evidence of active infection (Table 3). Most women had an RDT done because of asymptomatic anaemia (n = 126, 79.8 %), and 13 % (n = 21/158) were positive for *P. falciparum* by RDT. RDT on peripheral blood failed to detect more than half of the women with active placental infection, as did LM and qPCR (Table 3). Because most infections at delivery were asymptomatic, and sample size was small, the analysis was not further stratified by testing criteria. Symptomatic women were twice as likely to have active placental infection compared to those who were tested for anaemia alone (symptomatic 30.6 % [11/36]; anaemia only 16.4 % [20/122], *P* = 0.060).

**Table 3:** RDT, LM and qPCR of peripheral blood and detection active *P. falciparum* placental infection on histology (n = 158)

	Placental infection (histology)		Sensitivity (%) (95 % CI)	Specificity (%) (95 % CI)	PPV (%) (95 % CI)	NPV (%) (95 % CI)
	Positive (n = 31)	Negative (n = 127)				
RDT						
Positive (n = 21)	14	7	45.2 (27.3, 64.0)	94.5 (89.0, 97.8)	66.7 (43.0, 85.4)	87.6 (80.9, 92.6)
Negative (n = 137)	17	117				
LM						
Positive (n = 16)	14	2	45.2 (27.3, 64.0)	98.4 (94.4, 99.8)	87.5 (61.7, 98.4)	88.0 (81.5, 92.9)
Negative (n = 142)	17	125				
qPCR						
Positive (n = 15)	13	2	41.9 (24.5, 60.9)	98.4 (94.4, 99.8)	86.7 (59.5, 98.3)	87.4 (80.8, 92.4)
Negative (n = 143)	18	125				
LM + RDT						
Positive (n = 25)	17	8	54.8 (36.0, 72.7)	93.7 (88.0, 97.2)	68.0 (46.5, 85.1)	89.5 (83.0, 94.1)
Negative (n = 133)	14	119				

Performance characteristics of RDT and LM for detection of active placental malaria on placental histology. *RDT* rapid diagnostic test, *LM* light microscopy, *qPCR* real-time polymerase chain reaction, *PPV* positive predictive value, *NPV* negative predictive value. Sensitivities and specificities of RDT, LM and qPCR were not statistically different (all *P* > 0.05, Liddell’s exact test)

## Discussion

The performance characteristics and utility of an HRP-2/pLDH combination RDT to detect parasitaemia in pregnant women in coastal PNG were evaluated as part of a clinical trial providing insecticide-treated bed nets and at least one dose of IPTp. RDTs were undertaken amongst trial participants presenting with anaemia or symptoms suggestive of malaria only. In this context RDT failed to diagnose about half of peripheral and placental *P. falciparum* infections detected by qPCR and histology, respectively. In addition, RDT missed one-third of infections (any species) detected by LM, falling well below the WHO performance criterion of 90 % sensitivity, considered to indicate adequate performance [17]. RDT performance varied with screening criteria and malaria species: sensitivity to detect *P. falciparum* was higher amongst symptomatic women, and poorer for asymptomatic anaemic women, or in women infected with *P. vivax*. In line with previous findings [41], RDTs did not outperform LM to detect PCR-confirmed peripheral *P. falciparum* and *P. vivax* infections during pregnancy.

The overall poor performance of an HRP-2/pLDH combination RDT to detect *P. falciparum* as detected by qPCR suggests limited utility as a malaria screening tool in pregnancy in PNG. Poor performance observed in this study may be explained by low sensitivity to detect low density parasitaemias, although the median parasite densities for *P. falciparum* exceeded the minimum detection threshold documented in laboratory testing of the RDT test (>100/µL) [44] and those specified by the WHO (>200/µL) in all groups [45]. Not all previous studies evaluating HRP-2-based RDTs demonstrated inferiority of peripheral blood RDT compared to LM. In asymptomatic pregnant Congolese women RDT was better at diagnosing peripheral infection detected by nested PCR compared to LM [43]. In Uganda, peripheral blood HRP-2-RDT and LM detected most placental infections (histology), and performed best when combined [41]. The differences in findings may be explained by differences in parasite densities, HRP-2-RDT brand, as well as laboratory procedures and assays used.

It is well established that in areas with moderate-to-high transmission intensity the majority of parasitaemic pregnant women will be asymptomatic. As such, RDT performance to detect infection in asymptomatic women is critical. RDTs had poor sensitivity for detection of peripheral *P. falciparum* parasitaemia amongst asymptomatic women with anaemia (Hb <90 g/L). This finding has implications at a number of levels.

The suboptimal performance of the HRP-2/pLDH RDT amongst asymptomatic anaemic women suggests that it may not be appropriate to use this diagnostic tool as a component of IST. Although the association between infections missed by RDT and adverse mother-infant outcomes could not be evaluated in the present study, a recent study conducted in Benin provides compelling evidence of the detrimental effect of submicroscopic infections on increased risk of maternal anaemia and low birth weight in primigravidae [14]. Second, malarial infection is a well-established risk factor for gestational anaemia [1], and preliminary data from the parent trial indicates the same [46]. This means that the testing criteria of anaemia should bias towards parasitaemia. Future studies in unselected women, including those without symptoms and a normal Hb, would be expected to show similar, or even poorer sensitivity of RDTs for detection of parasitaemia. Third, anaemia is common in pregnant and non-pregnant individuals in PNG [29], yet in the present study the proportion of anaemic women with parasitaemia was comparatively low. This means anaemia is not a valuable clinical criteria to improve RDT performance, and calls for further research into the causes of anaemia in pregnant women in PNG.

Taken together these findings suggest that further optimization of existing RDTs, or the development of novel, molecular approaches such as loop-mediated isothermal amplification (LAMP) [47, 48], to accurately screen for infection in antenatal clinics in malaria-endemic areas is urgently required. Until this is achieved, and more is known about the association between subpatent infection and adverse pregnancy outcomes in the region, this study supports the current IPTp policy in coastal PNG of three doses of SP, rather than ISTp.

RDT performance was better in women screened because of malaria symptoms than for asymptomatic anaemia, possibly because of the higher parasite densities in symptomatic women. This observation is in keeping with previous research that demonstrates near-adequate performance in febrile pregnant women in Africa [41]. The present testing criteria were based on malaria symptoms described in hyperendemic areas in Africa [30, 31]. Further optimization of testing criteria specific for PNG may improve RDT performance, however complex clinical algorithms to guide RDT testing may be impractical in a resource-limited setting like PNG. In addition, the prevalence of malaria in pregnant women was lower than previously observed [28, 36], probably because of the intensified malaria control strategies during the study period [49, 50]. If changes in malaria prevalence and immunity to malaria in pregnancy continue in the region [28], an increased number of symptomatic infections are anticipated, and on-going studies of the usefulness of RDTs to identify malaria in pregnancy will be required. The majority of women with symptoms did not appear to have malaria; the underlying causes of their symptoms are unknown.

At delivery, all measures of parasitaemia in peripheral blood significantly underestimated active placental malaria by histology, probably due to occult placental sequestration. This supports a previous study by Mayor et al. [5], who show that HRP-2-based RDTs fail to detect the majority of placental infections confirmed by qPCR. Here, qPCR of placental blood detected more infections than histology, and was used hence as the reference standard: performance of the HRP-2/RDT against placental blood PCR was not evaluated in this cohort. In the Congo HRP-2 RDT were useful for the detection of placental infection (according to histology) when used in combination and in presence of fever [41], however the combination of RDT and LM results did not further enhance performance during pregnancy in this study (Table 3), and the small sample size of delivery episodes precludes a meaningful sub-analysis by test criteria. Unlike African-based reports [11], the present study suggests there is insufficient evidence to support the use of RDTs for the diagnosis of placental malaria in PNG.

The few studies to evaluate RDT for *P. vivax* infection in non-pregnant individuals report varying performance [19, 20, 44]. The prevalence of *P. vivax* infections was lower than expected, and it was a rare cause of clinical malaria in the present study. The limited data show that RDT or LM missed nearly three quarters of infections detected by PCR. This suggests it is not a good screening tool for *P. vivax* infection in pregnancy. Future studies including PCR data to differentiate potential *P. ovale* and *P. malariae* infections, which are present but rare [36, 51], are required for a more definite and comprehensive RDT performance analysis for the detection of *P. vivax* infection.

Although this study is one the largest to assess the usefulness of an HRP-2/pLDH RDT in pregnancy in the Asia–Pacific region, it is subject to a number of limitations. First, the findings are restricted to pregnant women presenting with moderate to severe anaemia and/or non-specific clinical malaria symptoms: RDT performance amongst asymptomatic pregnant women without anaemia is unknown. Second, only a subset of women were tested at delivery and had placental biopsies taken: the small sample size increases the risk of random error and does not allow definitive conclusions regarding the performance of HRP-2/pLDH-RDT to detect placental infection. In addition, histology was not evaluated against placental blood qPCR. Third, the low prevalence of infection overall limits extrapolation of findings to areas of higher malaria transmission, but does reflect much of the malaria epidemiology across the Asia–Pacific region. Fourth, this study was conducted within the context of an IPTp trial in women sleeping under insecticide treated bet nets. Although most RDT screening events took place at enrolment, before women received any treatment, interpretation of the study data is limited to this context. Moreover, qPCR species-correction of five infection episodes may have biased the diagnostic performance of LM. While operator error cannot be excluded at clinical or laboratory level, clinical training, refresher training and competency assessment were provided throughout the study, rendering operator factors as an unlikely cause of poor RDT performance. Microscopy and molecular diagnostic facilities at the PNG Institute of Medical Research undergo regular external quality control and operate at the highest standard. Finally, the low prevalence of malaria in this cohort and the study design did not permit an evaluation of the impact of missed infections on pregnancy outcomes.

## Conclusion

At the current level of transmission in lowland coastal PNG, HRP-2/pLDH RDTs may be used to diagnose peripheral *P. falciparum* infections in symptomatic pregnant women, but should not be employed for screening amongst asymptomatic (anaemic) women. A more sensitive tool is required to effectively detect and guide treatment of all women with *Plasmodium spp* infections. The potential adverse impact of infections undetected by RDT or LM on pregnancy outcomes needs further evaluation. Until more sensitive point of care diagnostics are available, IPTp with SP is likely to remain the optimal strategy for malaria prevention in pregnancy in PNG.

## Additional files

10.1186/s12936-015-0927-5 Negative qPCR and LM results amongst HRP2-band positive RDT screening episodes.
10.1186/s12936-015-0927-5Comparison of HRP2/pLDH RDT (and light microscopy) against qPCR (reference) for detection of *P. falciparum* in peripheral blood, by trimester and gravidity.
