# Supplementary material for: Accuracy of an HRP-2/panLDH rapid diagnostic test to detect peripheral and placental Plasmodium falciparum infection in Papua New Guinean women with anaemia or suspected malaria
Source: Malar J. 2015 Oct 19;14:412. doi: 10.1186/s12936-015-0927-5 (PMC4617889; doi:10.1186/s12936-015-0927-5)
Supplement: Supplementary file 1 — 10.1186/s12936-015-0927-5 Negative qPCR and LM results amongst HRP2-band positive RDT screening episodes. [file 12936_2015_927_MOESM1_ESM.docx]

**Supplementary Table** **2** Comparison of HRP2/pLDH RDT (and light microscopy) against qPCR (reference) for detection of *P. falciparum* in peripheral blood, by trimester (according to symphysis-pubis fundal height) and gravidity.

| **Category/test** | | **Prevalence by qPCR**  **(95% CI)** | **Sensitivity**  **(95% CI)** | **Specificity**  **(95% CI)** | **PPV**  **(95% CI)** | **NPV**  **(95% CI)** | **% (n/total) of women tested for symptoms suggestive of malaria** |
| --- | --- | --- | --- | --- | --- | --- | --- |
| *Gravidity* | |  |  |  |  |  |  |
| Primigravida | |  |  |  |  |  | 29.0 (159/548) |
|  | RDT | 18.0 (15.0, 21.9) | 42.6 (32.8, 52.8) | 96.6 (94.5, 98.1) | 74.1 (61.0, 84.7) | 88.2 (85.0, 90.0) |  |
|  | LM |  | 65.3 (55.2, 74.5) | 97.1 (95.1, 98.4) | 83.5 (73.5, 90.9) | 92.5 (89.8, 94.7) |  |
| Secundigravida | |  |  |  |  |  | 23.5 (53/226) |
|  | RDT | 16.0 (11.0, 21.4) | 47.2 (30.4, 64.5) | 95.8 (91.9, 98.2) | 68.0 (46.5, 85.1) | 90.5 (85.6, 94.2) |  |
|  | LM |  | 66.7 (49.0, 81.4) | 97.4 (94.0, 99.1) | 82.8 (64.2, 94.2) | 93.9 (89.6, 96.8) |  |
| Multigravida | |  |  |  |  |  | 24.7 (96/388) |
|  | RDT | 8.8 (6.1, 12.0) | 52.9 (35.1, 70.2) | 96.3 (93.8, 98.0) | 58.1 (39.1, 75.5) | 95.5 (92.8, 97.4) |  |
|  | LM |  | 55.9 (37.9, 72.8) | 98.3 (96.3, 99.4) | 76.0 (54.9, 90.6) | 95.9 (93.3, 97.7) |  |
|  | |  |  |  |  |  |  |
| *Trimester at screening episode (n = 1,160)* | |  |  |  |  |  |  |
| Second trimester* | |  |  |  |  |  | 24.3 (163/670) |
|  | RDT | 17.0 (14.0, 19.8) | 31.3 (22.8, 40.7) | 95.7 (93.7, 97.2) | 59.3 (45.7, 71.9) | 87.4 (84.5, 89.9) |  |
|  | LM |  | 58.9 (49.2, 68.1) | 96.8 (94.9, 98.1) | 78.6 (68.3, 86.8) | 92.2 (89.7, 94.2) |  |
|  | |  |  |  |  |  |  |
| *Third trimester* | |  |  |  |  |  | 29.4 (144/490) |
|  | RDT | 12.0 (9.3, 15.3) | 72.9 (59.7, 83.6) | 97.2 (95.2, 98.6) | 78.2 (65.0, 88.2) | 96.3 (94.1, 97.9) |  |
|  | LM |  | 72.9 (59.7, 83.6) | 98.6 (97.0, 99.5) | 87.8 (75.2, 95.4) | 96.4 (94.2, 97.9) |  |

Performance characteristics. **Note.** Trimester was estimated according to symphysis-pubis fundal height.

RDT, rapid diagnostic test; LM, light microscopy; qPCR, real-time polymerase chain reaction; PPV, positive predictive value; NPV, negative predictive value. * Includes 18 screening episodes undertaken in the first trimester of pregnancy
