# Supplementary material for: Accuracy of an HRP-2/panLDH rapid diagnostic test to detect peripheral and placental Plasmodium falciparum infection in Papua New Guinean women with anaemia or suspected malaria
Source: Malar J. 2015 Oct 19;14:412. doi: 10.1186/s12936-015-0927-5 (PMC4617889; doi:10.1186/s12936-015-0927-5)
Supplement: Supplementary file 2 — 10.1186/s12936-015-0927-5Comparison of HRP2/pLDH RDT (and light microscopy) against qPCR (reference) for detection of P. falciparum in peripheral blood, by trimester and gravidity. [file 12936_2015_927_MOESM2_ESM.docx]

**Table S1** qPCR and LM results amongst HRP2-band positive RDT screening episodes that were either negative by LM or qPCR (*P. falciparum*)

| **Scenario** | **Result** | | **Comments** |
| --- | --- | --- | --- |
| HRP2 positive, LM negative for  *Plasmodium falciparum* | *qPCR* | |  |
|  | Positive | Negative | Mean threshold cycle: 28.7  Median: 28.8  Range: 21.3 – 39.0 |
| 29 | 7 (24.1) | 22 (75.6) |  |
|  |  | |  |
| HRP2 positive, qPCR negative for  *Plasmodium falciparum* | *Light microscopy* | |  |
|  | Positive | Negative | Mean parasitaemia: 637*  Median: 379  Range: 22 – 11,492 |
| 34 | 12 (35.3) | 22 (64.7) |  |

**Note**. HRP-2, histidine-rich protein-2; LM, light microscopy; qPCR, real-time polymerase chain reaction. Amongst 22 women with a positive test episode for *P. falciparum* (HRP-2) but who were negative for both LM and qPCR nine women were tested at enrolment (pre-trial intervention). Of them two stated having received antimalarials (unknown timing) in the index pregnancy prior to enrolment. The remaining 13 testing episodes took place after enrolment. Five of them were done amongst women who had antimalarial treatment 30 days or less before the testing episode. The remaining women had their last (documented) antimalarial treatment more than 78 days prior to the testing episode.

* Geometric mean
